# Bacteriophage as effective decolonising agent for elimination of MRSA from anterior nares of BALB/c mice

**DOI:** 10.1186/s12866-014-0212-8

**Published:** 2014-08-12

**Authors:** Sanjay Chhibber, Paridhi Gupta, Sandeep Kaur

**Affiliations:** 1 Department of Microbiology, Basic Medical Sciences (BMS) Block, Panjab University, Chandigarh 160014, India

**Keywords:** Nasal colonisation, S. aureus, Nasal epithelial cells, Adherence, Invasion

## Abstract

**Background:**

Nasal carriers not only pose serious threat to themselves but also to the community by playing an active role in the dissemination of serious and life threatening *S. aureus* especially MRSA strains. The present study focuses on the use of broad spectrum lytic phage as decolonising agent. In addition, the combined use of lytic phage with mupirocin has also been investigated as an effective decolonising regimen. The effect of phage on the adherence, invasion and cytotoxic effect of MRSA strains on nasal epithelial cells was studied in an *ex-vivo* model of cultured murine nasal epithelial cells. This was followed by demonstration of therapeutic potential of phage along with mupirocin in decolonising the nares of BALB/c mice using a nasal model of MRSA colonisation.

**Results:**

Phage was able to significantly reduce the *in vitro* adherence, invasion and cytotoxicity of MRSA 43300 as well as other clinical MRSA strains on murine nasal epithelial cells as compared to untreated control. Also, the frequency of emergence of spontaneous mutants decreased to negligible levels when both the agents (phage and mupirocin) were used together.

**Conclusion:**

Phage MR-10, given along with mupirocin showed an additive effect and the combination was able to effectively eradicate the colonising MRSA population from the nares of mice by day 5.

## Background

Approximately 20% of healthy adults are persistent nasal carriers of *S. aureus* and 60% harbour it intermittently. Such carriers have been shown to participate in the epidemiology and pathogenesis of *S. aureus* infections and are a potential source of outbreaks especially in hospital settings [1],[2]. Nasal carriers are at an increased risk of acquiring surgical site infections, foreign body infections and bacteremias [3],[4]. Although nasal colonisation with MRSA is low but such carriers are a major threat factor for themselves (through auto-infection/endogenous source) as well as can disseminate these highly resistant strains that pose serious difficulty in treatment thereafter.

The current treatment strategies for nasal decolonisation rely on the use of topical antibiotics such as bacitracin, fusidic acid, ciprofloxacin, rifampicin [5]. However, emergence of resistant strains has led to treatment failures. Mupirocin is another potent anti-MRSA agent which has been found to be effective in decolonising the nares. Long term studies have however, shown that there is an initial clearance of bacteria from nares following mupirocin treatment but re-colonization takes place after 3 months [6],[7]. The rapid emergence of resistance to mupirocin therefore calls for search for alternative options. Phage therapy has been shown to be a potential alternative treatment for treating various *S. aureus* infections [8]-[13]. Hence, an alternative or supplement to antibiotic therapy, is the use of bacterial viruses (phage/bacteriophage) to target MRSA colonisation in the anterior nares of the affected population.

However, there is comparatively limited work published on the use of phages as nasal decolonising agents as compared to their proven therapeutic potential in other infections. Moreover, the combined application of phage and antibiotic in eliminating the nasal load of *S. aureus* has not been looked into earlier studies. Combination therapy (use of two different agents) represents an attractive option for nasal decolonisation due to its ability to check emergence of resistant mutants [13],[14].

## Methods

### Ethical statement

The experimental protocols were approved by the Institutional Animal Ethics Committee of the Panjab University, Chandigarh, India and performed in accordance with the guidelines of Committee for the Purpose of Control and Supervision of Experiments on Animals (CPCSEA), Government of India, on animal experimentation. All efforts were made to minimize the suffering of animals.

### Bacterial strains and phage used

*S. aureus* ATCC 43300(MRSA) and *S. aureus* ATCC 29213(MSSA) from ATCC, Mannasse, USA were used in this study. These two strains were used to study the bacterial adherence, invasion and cytotoxicity on cultured murine epithelial cells. However, *S. aureus* 43300 was used to establish the nasal colonisation in BALB/c mice. Clinical isolates of *S. aureus* were procured from Post-graduate Institute of Medical Education and Research (PGIMER), Chandigarh, India. The strains were isolated from clinical specimens (nasal screening swabs, blood, pus, soft tissue, wound swabs, respiratory samples and body fluids) collected from both in-patient and as out-patient subjects. The strains were identified on the basis of Gram reaction, growth on mannitol salt agar (MSA), catalase activity, and coagulase test. Methicillin resistance was determined using cefoxitin disk on Mueller-Hinton agar (Oxoid) followed by determination of MICs of oxacillin for these strains as recommended by Clinical and Laboratory Standards Institute (CLSI) [15]. A total of thirty four MRSA isolates were selected, numbered sequentially as MRSA 01 to MRSA 34 (clearly depicting their source) and stored in glycerol at −80°C. These strains were used for determining the lytic spectrum/host range of the isolated phage.

*S. aureus* specific bacteriophage, MR-10, which had been isolated and characterized in our laboratory was used in the present study [13]. This phage was selected as it showed a broad host range against four standard strains of *S. aureus* [*S. aureus* ATCC 43300(MRSA), *S. aureus* ATCC 29213(MSSA), *S. aureus* ATCC 25923(MSSA) and *S. aureus* ATCC 33591(MRSA)] as well as was effective against 32/34 clinical MRSA isolates (data depicting the host range of MR-10 is included in Additional file 1: Table S1).

### Animals used

BALB/c female mice, 4–6 weeks old weighing 20–25 g were used in this study. The animals were obtained from Central Animal House, Panjab University, Chandigarh. The animals were kept in well aerated rooms and given antibiotic free diet (Hindustan Lever, Mumbai) and water ad libitum.

### Isolation and culturing of murine nasal epithelial cells (NEC)

This was performed according to the method of Grubb *et al*. [16]. Nasal septum was dissected from five mice and washed with Dulbecco modified Eagles Medium (DMEM) with 100 μg/ml streptomycin. The septum was homogenized and centrifuged at 2000 rpm for 10 min. The nasal tissue was re-suspended in dissociation medium (10 mM HEPES- streptomycin-DMEM) overnight at 4°C. Next day, the tissue suspension was again centrifuged and suspended in isolation media (145 mM NaCl, 4.5 mM KCl, 10 mM glucose, 10 mM HEPES) and kept at 37°C for 2 hours. The tissue was then teased gently using 26G needle to form single cell preparation. The cell suspension was passed through cell strainer (100 μ Nylon; BD) and given washings thrice and finally suspended in DMEM. Cells were viewed under phase contrast (Olympus, 40×) and counted using trypan blue staining to determine cell viability in a haemocytometer.1 ml of 10^5^ cells/ml was seeded in each well of 12 well plate and incubated at 37°C in 5%CO_2_. The cells were monitored each day for cell density and increase in cell size, using crystal violet staining of smears prepared from the cells.

### Preparation of NEC and bacteria inoculum for adherence, invasion and cytotoxicity assay

Cells obtained on day 5 of culturing were aspirated from their respective wells and transferred to microfuge tube. Cells were centrifuged at 1800 rpm for 10 min at 4°C. The pellet so obtained was washed twice with PBS (pH 7.2) and finally re-suspended in DMEM. Cells were stained using trypan blue and counted in haemocytometer. An average of 10^6^ nasal cells/ml were used for adherence assay. *S. aureus* ATCC 43300(MRSA), *S. aureus* ATCC 29213(MSSA) and five different clinical MRSA isolates (for which phage MR-10 showed activity) were used in the adherence, invasion and cytotoxicity assay. Single colony of bacteria was inoculated in sterile BHI broth and incubated overnight. Next day, cells were harvested by centrifugation at 10,000 rpm for 15 minutes at 4°C. The pellet so obtained was washed twice with sterile normal saline (0.85%). The final pellet obtained was suspended in normal saline and its O.D(600 nm) adjusted so as to obtain cell density corresponding to 10^8^ CFU/ml. This was confirmed by plating on nutrient agar plates.

### Adherence assay

Washed nasal epithelial cells, re-suspended in DMEM were seeded in 12 well plate. Bacterial suspension (corresponding to 1 × 10^8^ CFU/ml) was added to obtain a ratio of 10:1(Bacteria : nasal epithelial cells). Following 3 h of incubation at 37°C in 5% CO_2_, the inoculum was removed and the epithelial cells were washed thrice with PBS by centrifugation at 1800 rpm for 10 min at 4°C to remove non associated bacteria. (Note: Supernatant after each wash was plated on nutrient gar plates and after third wash, there was complete removal of the non-adhered bacterial cells). The cells were then treated with lysis solution (0.025% trypsin and 1% tween 20 in PBS) for 30 min at 37°C. Total number of associated bacteria (T) (adherent and invaded) was assessed by plating suitable dilutions of the cell suspension on nutrient agar plates. The final results were expressed as% adherence. Suitable control containing only nasal epithelial cells with no added bacteria was also processed in the same way to check for sterility throughout the experiment.

### Invasion assay

The gentamicin survival assay was performed as per the method of El-Housseiny *et al*. [17] in order to determine the number of invaded bacteria. After the addition of bacteria (10:1) to the seeded NEC, the plate was incubated for 3 h at 37°C in 5% CO_2_. It is essential to remove adherent as well as extracellular bacteria in order to determine the invaded population. For this, gentamicin solution was added to all the wells at a concentration of 25 μg/ml and the plate was incubated for 1 h at 37°C in 5% CO_2_ to kill the extracellular bacteria (Note : this concentration was based on the MIC value of gentamycin determined against MRSA 43300 which was 16 μg/ml. In addition, after treatment with 25 μg/ml of gentamycin for 1 hour, the supernatant containing killed bacteria was plated out with complete killing (no colonies on incubation) observed). Finally, the epithelial cells were washed thrice with PBS by centrifugation at 1800 rpm for 10 min at 4°C to remove non associated bacteria. The cells were re-suspended in DMEM and then treated with lysis solution (0.025% trypsin and 1% tween 20 in PBS) for 30 minutes at 37°C in 5% CO_2_. The cell suspension so obtained was suitably diluted and plated on nutrient agar plates. This bacterial count so obtained represented the number of invaded bacteria **(I)**.

The difference between the total number of associated bacteria **(T)** and the number of invaded bacteria **(I)** was taken as number of adhered bacteria = **(T-I)** CFU/ml. Results were expressed as % invasion and % adherence.

### Cytotoxicity assay

To determine the cytotoxic effect of *S. aureus* cells on NEC, (4,5-Dimethylthiazol-2-yl)-2,5-diphenyltetrazolium bromide (MTT) reduction assay was performed as per the method of Saliba *et al*. [18]. Washed nasal cells, re-suspended in DMEM were seeded in 12 well plate. After addition of bacteria (bacteria: NEC- 10:1), the plate was incubated for adherence to occur. After 6 h of incubation, gentamicin was added to the wells to kill the extracellular bacteria. To the washed cells, MTT was added (2 mg/ml in PBS) and incubated for 1 h at 37°C in 5% CO_2_. Supernatant was discarded and cells were treated with 100 μl of absolute ethanol to dissolve the formazan crystals and absorbance measured at 540 nm. The same procedure was repeated at 24 and 48 hours. Suitable control wells containing only epithelial cells without added bacteria were also processed in the same way at all time points. The percentage cytotoxicity was calculated using the following formula:

(1) $$\%\;\mathrm{Cytotoxicity}\;=\;\left[1‐\;\left(A_{540}\mathrm{of}\;\mathrm{test}\;\mathrm{well}/\;A_{540}\mathrm{of}\;\mathrm{control}\;\mathrm{well}\right)\;\times \;100\right]$$

### Effect of phage on bacterial adhesion, invasion and cytotoxicity on NEC

Washed nasal epithelial cells re-suspended in DMEM were seeded in 12 well plate. Bacterial suspension (corresponding to 1 × 10^8^ CFU/ml) was added to nasal epithelial cells (10:1). Following bacterial addition, phage was added at MOI-1 and 10, and the plate was incubated for 3 h at 37°C in 5% CO_2_. After incubation, the inoculum was removed and the epithelial cells were washed thrice with PBS by centrifugation at 1800 rpm for 10 min at 4°C to remove non associated bacteria. The cells were re-suspended in DMEM. The cells were then treated with lysis solution (0.025% trypsin and 1% tween 20 in PBS) for 30 min at 37°C in 5% CO_2_. Total number of associated bacteria (T) (adherent and invaded) was assessed by plating suitable dilutions of the cell suspension on nutrient agar plates.

Similarly, for invasion assay, washed nasal epithelial cells were incubated with the respective bacterial suspension (corresponding to 1 × 10^8^ CFU/ml) and phage was added at MOI-1 and 10. The plate was incubated for 3 h at 37°C in 5% CO_2_. This was followed by addition of gentamicin solution (25 μg/ml) to kill the extracellular bacteria. The epithelial cells were washed thrice with PBS to remove non associated bacteria and phage. The cells were re-suspended in DMEM, treated with lysis solution. The cell suspension so obtained was suitably diluted and plated on nutrient agar plates.

For cytotoxicity assay, washed nasal cells, re-suspended in DMEM were seeded in 12 well plate. After addition of bacteria (bacteria: NEC- 10:1), phage was added at MOI-1 and 10. The plate was incubated for different time intervals (6 h, 24 h and 48 h) at 37°C in 5% CO_2_. After the completion of respective time interval, gentamicin was added to the wells to kill the extracellular bacteria. After this step, same procedure was repeated as described under cytotoxicity assay.

### Appearance of bacteriophage insensitive mutant (BIM) and mupirocin resistant mutants

The frequency of spontaneous mutation in *S. aureus* 43300 on exposure to phage and mupirocin was determined. For BIM frequency, plaque assay was performed using an overnight culture of *S. aureus* 43300 containing known bacterial numbers and phage added at MOI-10 respectively. The plates were incubated overnight at 37°C. All resulting colonies were counted, and the BIM frequency was determined by dividing the number of surviving colonies by the original bacterial titer. Similarly, spontaneous mutation frequency for mupirocin was also determined at both 2 and 4 μg/ml according to the method of O’Neill *et al.*[19] using cation adjusted Mueller Hinton agar plates. The frequency of spontaneous mutation was determined by dividing the number of surviving colonies on selective plates by total number of colonies on non-selective plates after 48 hours of incubation.

Frequency of appearance of resistant mutants in presence of both phage (MOI-10) and mupirocin together was determined by performing the plaque assay on selective plates with 2 and 4 μg/ml of mupirocin.

### Antibiotic susceptibility of bacteria isolated from murine nares

Three independent colonies were regularly isolated (data shown in Additional file 1: Table S2) from the nares of randomly selected male BALB/c mice in six independent experiments. These were referred to as NS-1, NS-2 and NS-3. For evaluating the bacterial load of *S. aureus* 43300 on different days post-colonisation in the murine nasal colonisation model described below, a selective media allowing the growth of only *S. aureus* 43300 without interference from the nasal flora was needed. Hence, nutrient agar plates with different concentrations of ampicillin (4, 8, 16, 20 and 32 μg/ml) were prepared. All the nasal isolates (NS-1, NS-2, NS-3, *S. aureus* 29213 as well as *S. aureus* 43300) were spread plated respectively. Nutrient agar plates with no antibiotic were used as control. All the plates were incubated for 24 h at 37°C. Next day, growth was observed on plates and the ampicillin concentration showing complete inhibition of growth (no colonies on selective plates) was noted. Ampicillin at a concentration of ≥16 μg/ml completely inhibited the growth of NS-1, NS-2 and NS-3 however MRSA 43300 growth was inhibited at 32 μg/ml. Hence, a dose of 20 μg/ml ampicillin was selected to be added to nutrient agar for preparing selective plates which allowed the growth of MRSA 43300 colonies only with no interference from nasal flora strains.

### Nasal carriage model of *S. aureus* 43300

*S. aureus* 43300 was cultivated for 24 h at 37°C in brain heart infusion broth. Next day, cells were pelleted and washed twice with phosphate-buffered saline (PBS). Bacterial suspension prepared in PBS was adjusted at 600 nm so as to achieve a cell density corresponding to a range of bacteria inoculums (10^5^,10^6^ and 10^7^ CFU/ml). The number of CFU/ml was confirmed by quantitative plate count. Mice were grouped randomly into three groups (N = 3) with twenty mice (n = 20) per group. For intranasal instillation, a 50 μl inoculum of respective bacterial dose was instilled into the nasal opening while holding the mice upright. The mouse was held upright for at least 2 minutes to allow the mice to take the inoculum with minimum loss. After an interval of 48 hours, second dose of inoculum was again instilled into the nares of mice in the same way as described above. Four mice from each group were sacrificed on day 2, 5, 7, 10 and 12 post inoculum administrations. After disinfecting the nasal area with 70% alcohol, the nasal tissue was dissected from each mouse and washed twice in PBS (pH 7.2). The tissue was homogenized, and dilutions of the homogenates were plated on nutrient agar plates to evaluate total bacterial flora. The homogenate dilutions were also plated on nutrient agar plates containing ampicillin (20 μg/ml) so as to check the load of *S. aureus* 43300 colonised in the nasal tissue.

### Phage and mupirocin protection studies

Therapeutic potential of bacteriophage, MR-10 alone as well as in combination with mupirocin was evaluated for its ability to reduce the nasal carriage in BALB/c mice.

**Figure 1:**
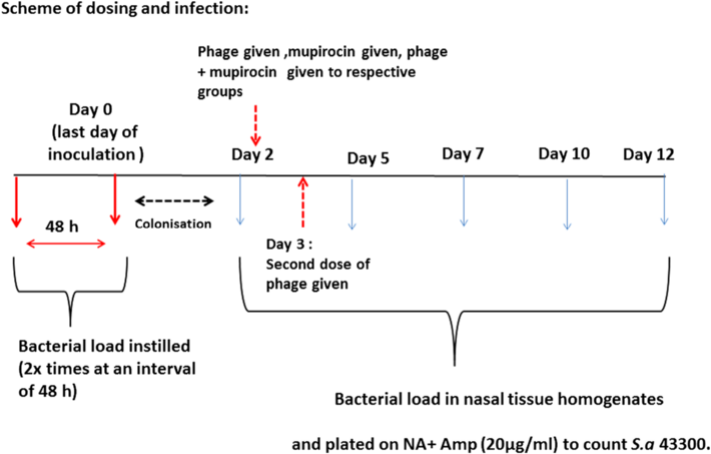
Schematic representation of the infection and treatment schedule followed for establishing nasal colonization model in BALB/c mice.

Male BALB/c mice were used and randomly divided into four groups (N = 4) with each group containing 20 mice each (n = 20). The infection and treatment schedule is depicted in Figure 1.

**Group 1:** Mice were administered *S. aureus* 43300(10^6^ CFU/ml) intranasally

**Group 2:** Mice were administered *S. aureus* 43300, left for a period of 48 hours to allow nasal colonisation followed by intranasal administration of 50 μl of phage (10^7^ PFU/ml) given twice (at an interval of 24 hours).

**Group 3:** Mice were administered *S. aureus* 43300, left for a period of 48 hours to allow nasal colonisation followed by intranasal administration of 50 μl of mupirocin (5 mg/kg dissolved in water; given once) the next day.

**Group 4:** Mice were administered *S. aureus* 43300, left for a period of 48 hours to allow nasal colonisation followed by intranasal administration of phage as well as mupirocin (5 mg/kg) the next day.

The parameters used to monitor colonization included a) Bacterial load (CFU/ml) in nares b) Phage counts in nares c) Nasal myeloperoxidase (MPO) levels and e) Histopathological examination

#### Nasal bacterial load

Four mice from each of group were taken and sacrificed on day 2, 5, 7, 10, 12 post treatment by cervical dislocation. The nasal region was wiped externally with 70% ethanol, nose was removed along with nasal bone. The entire nasal tissue was excised using sterile scissors and homogenized. The homogenates were plated quantitatively on nutrient agar containing 20 μg/ml of ampicillin to select *S. aureus* 43300 after overnight incubation at 37°C. Nasal homogenates were also processed to determine the phage titer by modified double layer agar method [20].

#### Myeloperoxidase (MPO) estimation

Mice from each group (same groups as those categorized for phage protection studies with 20 animals per group) were killed and their nasal tissue was excised and homogenised in 50 mM PBS (pH 7.4). Nasal samples were processed for MPO determination as per the method of Greenberger *et al.*[21]. The absorbance was read immediately at 490 nm over a period of 4 minutes. MPO was calculated as the change in optical density (O.D) x dilution factor (D.F).

#### Histopathological examination

Extent of injury caused by *S. aureus* and healing of the colonized mouse nose following therapy with phage or antibiotic was assessed on the basis of histopathological analysis of the injured and recovered nose according to the method of Brans *et al.*[22]. The sections were picked on separate slides, stained with hematoxylin and eosin (Hi-Media, Mumbai) and the slides then examined under a microscope to evaluate the extent of damage.

#### Statistical methods

The data is expressed as mean ± standard deviation of replicated values where indicated. The statistical significance of differences between groups was determined by Student’s *t*-test (two groups),one-way ANOVA followed by a Tukey test using Sigma Stat, Graph pad prism (Graph pad software, San Diego, CA). p value of less than 0.05 and 0.01 was considered statistically significant for a confidence interval of 95% and 99% respectively.

## Results

**Figure 2:**
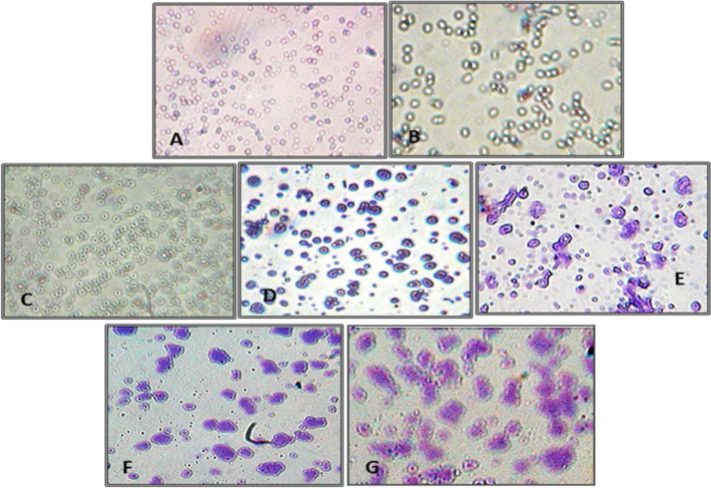
**Isolated murine nasal epithelial cells as observed under 40X Olympus light microscope on different days post-seeding. A)** and **B)** unstained and stained preparation of isolated single cells seen on the day of isolation **C)** unstained and **D)** stained preparation of cultured NEC on day 2 post seeding. Nucleus is clearly evident in all the cells **E)** and **F)** cells as seen on day 3 post seeding of different shapes and sizes and **G)** Polygonal shaped NEC as seen on day 5 with significant increase in size as well. These cells were harvested, counted and used for adherence and invasion studies.

The nasal epithelial cells were isolated from mouse nasal tissue and cultured at 37°C in presence of 5% CO_2_. Figure 2 depicts various stages of nasal epithelial cell (NEC) maturation on different days. The cells were observed as single cells at the time of isolation (Figure 2A and B). Thereafter, there was an increase in their size and density of the cells. Nucleus was clearly visible by day 2 and shape of the cells changed throughout the time of observation (Figure 2C and D). Day 3 onwards the cells differentiated into different shapes ranging from oval to round shape cells (Figure 2E and F). The cells obtained on day 5 (Figure 2G) were chosen for adherence studies as significant increase in size was attained by this time.

Since bacterial adherence is an essential step in the colonisation process of an organism, hence the percentage adherence of MRSA 43300 was studied using cultured NEC. Bacteria was added in order to obtain bacteria: nasal epithelial cell ratio of 1:1 and 10:1. The results presented in Table 1 show that bacteria exhibited high adherence (>50%) to nasal cells. The adherence was more (73.7%) when treated with higher number of bacterial cells i.e. 10:1. However, invasion of NEC was low, with only a maximum of 30% cells being invaded by the test bacteria. Similarly, cytotoxic damage inflicted by MRSA 43300 onto the cultured NEC was very low with an estimated value of just 3.6% and 9% at bacteria: NEC ratio of 1:1 and 10:1 respectively.

**Table 1:** **Effect of phage on adhesion, invasion and cytotoxicity of NEC by** ***S. aureus*** **43300**

**Treatments**	**Mean percent (%)**		
	**Adherence**	**Invasion**	**Cytotoxicity post 24 h**
**Control (Bacteria + NEC;1:1)**	58.6 ± 7.01	25 ± 3.73	3.6 ± 1.4
**Control (Bacteria + NEC;10:1)**	73.77 ± 7.8	31.90 ± 1.34	11.1 ± 0.7
**Phage (MOI-1)**	0.41 ± 0.202	0.0307 ± 0.012	0.21 ± 0.035
**Phage (MOI-10)**	0.0258 ± 0.005	No invasion	No cytotoxicity

### Effect of phage addition on bacterial adhesion, invasion and cytotoxicity of NEC

To demonstrate the effect of phage on the adherence and consecutively invasion and cytotoxicity of NEC by host bacteria, cultured NEC cells were processed in the same way with bacteria added in a ratio of 10:1. Following bacterial addition, phage was added at MOI-1 and MOI-10. Cells were then incubated for allowing adherence and invasion to occur.

From Table 1, it is evident that phage when added at MOI-1 and MOI-10 to *S. aureus* 43300, was able to significantly reduce (p < 0.05) all the three parameters as compared to untreated control. Only 0.4% of the bacterial cells showed adherence onto the nasal epithelial cells in presence of phage added at MOI-1. At higher MOI, adherence was reduced to negligible level. Similarly, almost minimal invasion and cytotoxic damage to NEC was observed with phage added at MOI-1. At higher phage concentration (MOI-10), the reduction in all the three parameters was highly significant (p < 0.01) and no invasion or cytotoxic damage was seen on NEC.

Table 2 depicts the adherence, invasion and cytotoxic damage of five different clinical MRSA strains denoted as CS-1 to CS-5(chosen at random) against which phage (MR-10) showed lytic activity. *S. aureus* 29213(MSSA) was also studied as an internal control. All the strains were found to adhere to cultured nasal epithelial cells in significant numbers (>60% adherence). The presence of phage significantly affected the adherence of all the strains (p < 0.01). Maximum invasion (33%) and cytotoxicity (14%) was observed with strain CS-3. The phage at MOI-1 was able to sixgnificantly decrease both the invasion and cytotoxic damage inflicted by all the clinical isolates. At higher MOI-10, no detectable invasion or cytotoxicity was observed.

**Table 2:** **Effect of phage on adhesion, invasion and cytotoxicity of NEC by additional clinical strains of** ***S. aureus*** **(MRSA)**

**Strains (Bacteria: NEC- 10:1)**	**Mean percent (%)**								
	**Adherence**			**Invasion**			**Cytotoxicity (24 h)**		
	**No phage**	**Phage (MOI-1)**	**Phage (MOI-10)**	**No phage**	**Phage (MOI-1)**	**Phage (MOI-10)**	**No phage**	**Phage (MOI-1)**	**Phage (MOI-10)**
*S. aureus* ATCC 43300 (MRSA)	73.7	0.41	0.025	31.9	0.031	No invasion	11.1	0.21	No cytotoxicity
*S. aureus* ATCC 29213 (MSSA)	76.8	0.51	0.034	18.4	0.034	No invasion	10.2	0.23	No cytotoxicity
*S. aureus* CS-1	68.4	0.37	0.066	28.1	0.06	No invasion	11.4	0.41	No cytotoxicity
*S. aureus* CS-2	62.5	0.32	0.074	25.4	0.064	No invasion	10.1	0.43	No cytotoxicity
*S. aureus* CS-3	74.8	0.45	0.084	33.3	0.078	No invasion	14.5	0.64	No cytotoxicity
*S. aureus* CS-4	70.4	0.34	0.081	30.4	0.072	No invasion	14	0.61	No cytotoxicity
*S. aureus* CS-5	72.1	0.33	0.075	32.8	0.066	No invasion	13.3	0.72	No cytotoxicity

(CS-1 to CS-5 : these are clinical strains (CS) of MRSA chosen at random to test for their adherence, invasion and cytotoxicity parameters on cultured murine NEC).

### Frequency of resistant mutant development

The frequency of emergence of resistant colonies using mupirocin was determined. The mupirocin resistant mutants *in vitro* appeared at a frequency of (7.1 ± 0.54) × 10^−6^ and (2.4 ± 0.14) × 10^−7^ at 2 and 4 μg/ml (2X and 4X MIC) respectively. The calculated bacteriophage insensitive mutant (BIM) frequency at multiplicity of infection (MOI) of 10 was comparatively higher with a value of (7.4 ± 0.21) × 10^−7^. However, when both the agents were used in combination, mutation rate was below detection limit (<10^−9^). The results clearly depict the advantage referred by combination treatment in decreasing the frequency of resistant mutant generation.

### Nasal colonisation model

A mouse model of nasal colonisation was established to assess the potential of phage to decolonize the nares of BALB/c mice from MRSA 43300. Three different inoculum doses (10^5^, 10^6^ and 10^7^ CFU/ml) of *S. aureus* 43300 were selected for establishing the organism in the nares of BALB/c mice. The inoculum of 10^5^ CFU/ml showed persistence of the organism in the nares only till day 5 post colonisation and the organism was cleared thereafter. At an inoculum dose of 10^6^ and 10^7^ CFU/ml, *S. aureus* 43300 persisted well till day 10 post colonisation with a load of 3.98 log CFU/ml (10^6^ CFU/ml) and 4.08 log CFU/ml (10^7^ CFU/ml) respectively and no counts observed on day 15 post colonisation. Since not much difference in the bacterial load of *S. aureus* 43300 in nares was observed with either of the two inoculum doses, hence 10^6^ CFU/ml was selected for establishing the nasal colonisation with *S. aureus* 43300 (Data depicting the nasal counts at all three different doses is shown in Additional file 1: Table S3).

### Bacterial load and phage titer

**Figure 3:**
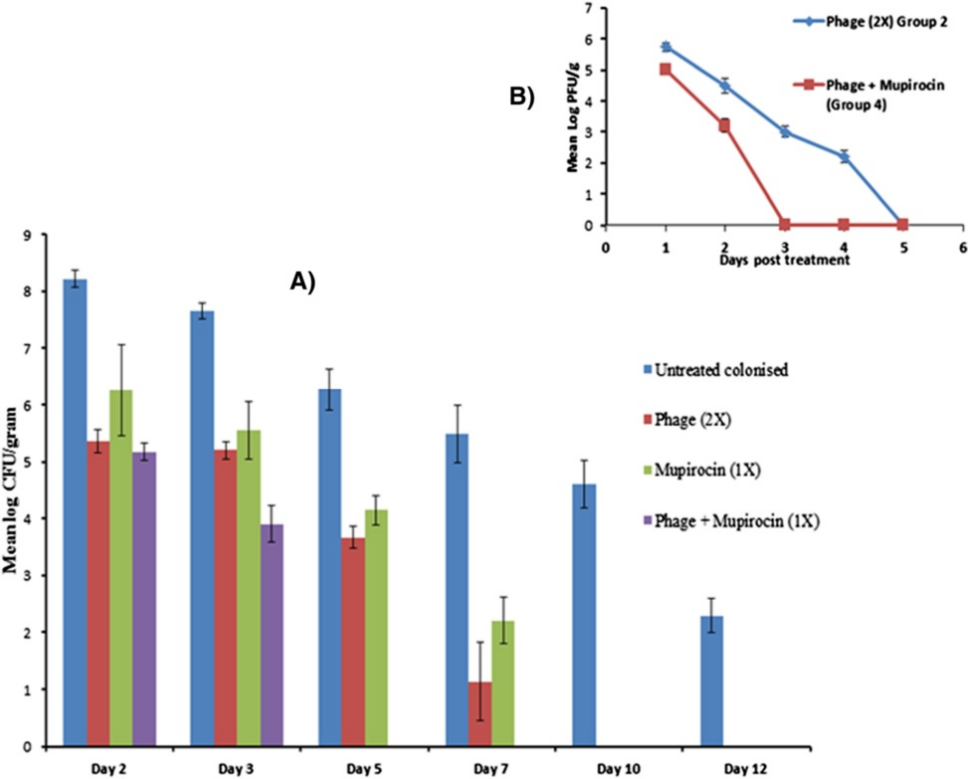
**Bacterial burden in terms of A) Mean log CFU/gram of mice tissue of*S. aureus*43300 following treatment of colonised nares with different anti-bacterial agents on different days post treatment; Phage counts in terms of B) Mean log PFU/g count in the anterior nares of mice belonging to group 2 and group 4 on various days post phage treatment.** Error bars represent the standard deviation.

The nasal load of *S. aureus* 43300 on different days post treatment is presented in Figure 3A. Mice administered with phage twice (group 2) showed significant reduction (p < 0.01) of 2.8 log-cycles in bacterial counts on day 2 itself. This was followed by further decrease in counts with 3.67 log CFU/g obtained on day 5 and minimal load of 1.14 log CFU/g seen on day 7. The nares became completely sterile as no growth of *S. aureus* 43300 was observed beyond day 7. Similarly, mupirocin given once (group 3) also showed significant reduction of ~2log cycles in comparison to control (group 1) on day 2. On day 7, minimal bacterial count of 2.21 log CFU/g was obtained after which there was complete clearance of *S. aureus* (Figure 3A).

The group receiving combined therapy (group 4) showed maximum reduction in bacterial load in the anterior nares with complete clearance of MRSA 43300 by day 5 itself The bacterial load was significantly reduced (p < 0.05) to 5.17 log CFU/g (~3 log-cycles) on day 2 and this decrease continued till day 3. By day 5, *S. aureus* 43300 was completely eradicated from the nasal tissue of BALB/c mice. The combined treatment option gave maximum protection against nasal colonisation by *S. aureus* 43300.

The animals receiving 2 doses of phage (10^7^ PFU/ml at an interval of 24 hours) showed a peak phage titre of 5.74 log PFU/g on day 2 (Figure 3B). Despite giving two doses of phage (10^7^ PFU/ml), only 10^5^ PFU/ml was present by day 2. A minimal phage titre (2.2 log PFU/g) was seen on day 7 with no plaques visible thereafter. In the co-therapy group, phage titres persisted only till day 3 (3.11 log PFU/g) and no plaque was seen on day 5.

### Myeloperoxidase assay

**Figure 4:**
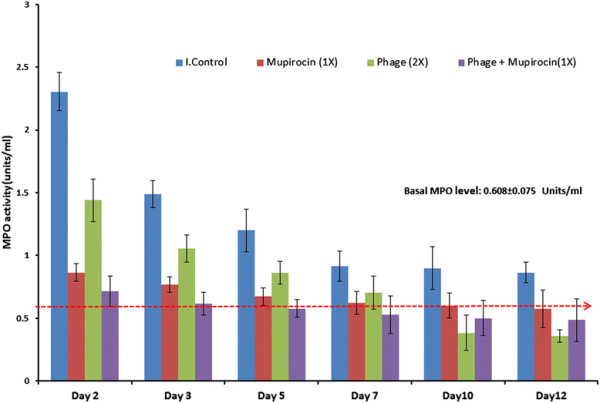
**Mean MPO activity (Units/ml) detected in the homogenates of nares of different groups of mice on different days post treatment.** Red dotted line represent the basal MPO activity as seen in healthy BALB/c mice (n = 4). Error bars represent standard deviation.

MPO levels were highest in untreated *S. aureus* ATCC 43300 colonised (group 1) animals on all days as shown in Figure 4. Peak MPO activity was seen on day 2 with further decrease on subsequent days. However, MPO levels were still higher on day 10 in this group than basal MPO levels (0.608 ± 0.075 units/ml) detected in the nares of normal healthy non-infected BALB/c mice (n = 3). A significant reduction (p < 0.05) in MPO activity (as compared to group 1) was seen in group 3 on all post-infection days. Similarly, phage treated group also showed decrease in MPO levels with peak (1.44 units/ml) seen on day 2 and 1.06 units/ml on day 3. By day 7, MPO levels almost similar to basal values were achieved. The group receiving combined therapy (group 4) showed minimal MPO levels on all days. MPO activity of 0.71 units/ml seen on day 2 accounted for a significant decrease of 69% (p < 0.05) in comparison to group 1.

### Histopathological examination

**Figure 5:**
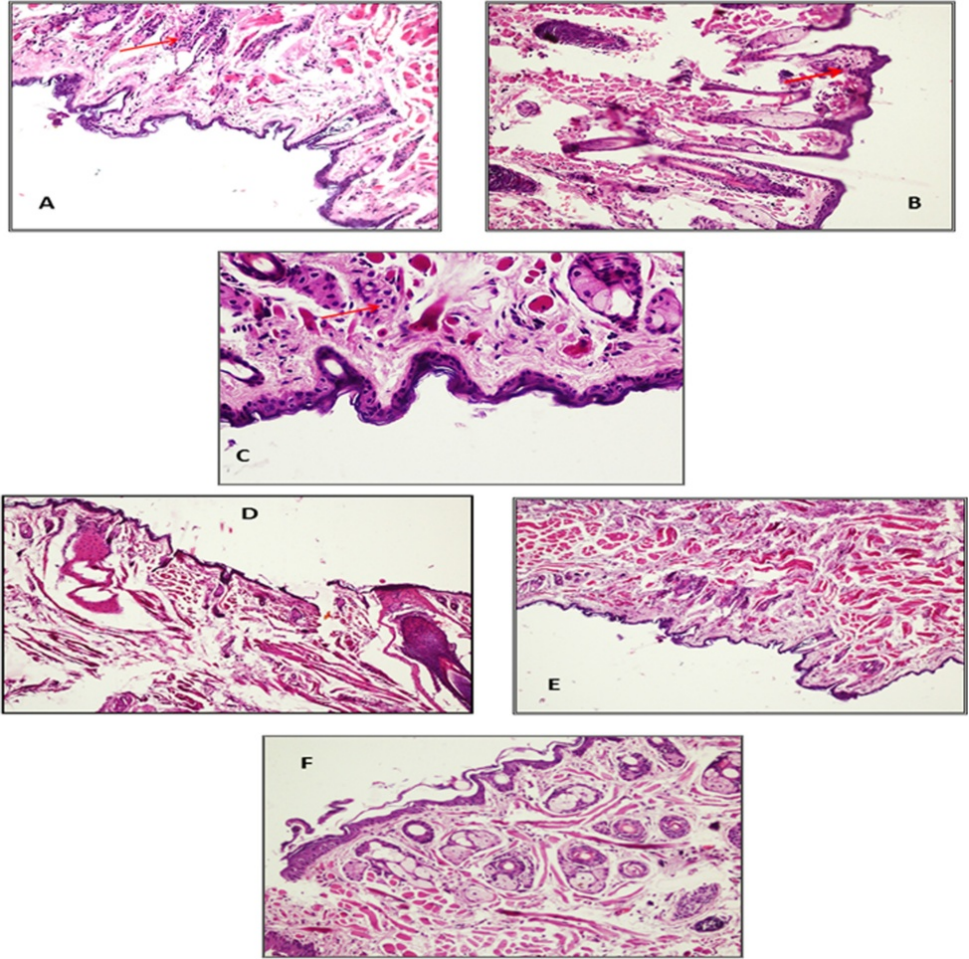
**Histopathological analysis showing. A)** Photo micrograph of skin tissue of nasal mucosa of untreated colonised mice on day 2 post colonisation showing mild inflammation with recruitment of few acute inflammatory cells(red arrows) (H and E 100X). **B)** and **C)** Photo micrograph of skin tissue of nasal mucosa of untreated colonised mice on day 5 post colonisation showing marked sub epithelial inflammation rich in neutrophils and plasma cells (H and E 100X and 200X). **D)** and **E)** Photo micrograph of skin tissue of nasal mucosa of phage treated (group 3) and Mupirocin treated(group 4) mice on day 5 post treatment showing mild infiltration in the sub epithelial lining (H and E 100X). **F)** Photo micrograph of skin tissue of nasal mucosa of mice receiving combined therapy (group 5) with nearly normal skin (H and E 100X).

As seen in Figure 5A, the nasal tissue of colonised untreated animals (group 1) on day 2 post colonisation, showed mild inflammation with recruitment of few acute inflammatory cells seen in the epidermis which was compressed by the collection of oedema fluids. Similarly, on day 5, the nasal mucosa of untreated colonised animals lined by squamous epithelium showed marked sub epithelial inflammation rich in neutrophils and plasma cells (Figure 5B and C). However, all the treated groups showed significantly reduced signs of inflammation. The nasal mucosa of phage treated group (group 2) (Figure 5D) on day 3 post treatment showed mild neutrophil and lymphoplasmatic infiltration in the sub epithelial lining with skin appearing nearly normal. Also, nasal mucosa of animals treated with mupirocin (group 3) (Figure 5E), showed small focus of mild inflammatory cells with skin appearing nearly normal. Minimum tissue inflammation was seen in nasal mucosa of animals receiving combined therapy (group 5) (Figure 5F) with no inflammation and skin appearing normal similar to nasal mucosa of healthy mice.

## Discussion

Mupirocin is considered as the best topical antibiotic available for gram positive bacteria [23],[24] and has been applied for nasal decolonisation since 1980s. However, emergence of bacterial resistance to mupirocin is fast rising leading to treatment failures and relapses [25]-[28]. In this study protection afforded by phage was therefore compared with mupirocin treatment. In addition, the additive effect if any, of the two agents as combination therapy in reducing/eliminating MRSA colonisation was also evaluated.

The first step in the colonisation by *S. aureus* is adherence to nasal epithelial cells and mucous membrane via bacterial cell surface moieties such as fibronectin binding protein, teichoic acid and adhesins [29]-[35]. In this study, the adherence and invasion pattern of MRSA 43300 on nasal cells was evaluated. Cultured murine nasal epithelial cells were used as substrates for studying the bacterial adherence. MRSA 43300 showed high adherence of 58.6 ± 7.01 and 73.77 ± 7.8% when added at a multiplicity of 1:1 and 10:1. The results confirmed the colonising ability of *S. aureus* MRSA 43300 onto the mouse nasal epithelium and its ability to survive in such cells for longer time. Additional five clinical MRSA isolates tested for their adherence ability also showed high adherence to murine nasal cells ranging from 62% to 75%.

*S. aureus* has the ability to invade the epithelial and endothelial cells, osteoblasts, fibroblasts, and human embryonic kidney cell lines [36]-[41]. These intracellular reservoirs of *S. aureus* possibly protect the bacteria from extracellular host defense mechanisms and antimicrobial treatment instilled for their elimination. This intracellular residency is now considered as one of the reasons of possible long term nasal carriage and persistence seen among chronic nasal carriers [40],[42]. Invasion of the epithelium by *S. aureus* and intracellular localisation of bacteria in the nasal epithelial cells *in vitro* has been demonstrated by Sachse *et al*. [43]. The presence of heavily infected foci of intracellular *S. aureus* in nasal epithelium cells was demonstrated by inverted confocal laser scan fluorescence and electron microscopy [44]. This was the first *in vivo* evidence of existence of internalized *S. aureus* in nasal carriers. The invasion of *S. aureus* is primarily promoted by fibronectin-binding proteins and integrin-mediated invasion of *S. aureus* into nonprofessional phagocytes has also been demonstrated [36]-[39],[45]-[48]. The ability of MRSA 43300 to invade the nasal epithelial cells in this study is supported by the fact that *S. aureus* ATCC 43300 posesses the *fnbB gene* which mediates invasion and thus 30% of the adhered population invaded the nasal epithelial cells. Cytotoxicity data revealed that this strain inflicted low(<10%) cytotoxicity post 24 hours of adherence. These results suggest that the bacteria posed little damage to the epithelial cells which infact may be beneficial for their long term survival within the host tissue.

The effect of phage on the adherence and invasion pattern of MRSA 43300 was determined using the *in vitro* model of cultured murine nasal epithelial cells. Phage at both the MOI (1, 10) was able to show highly significant reduction in all the three parameters as compared to untreated control. A pronounced decrease in the number of adhered bacterial population with negligible invasion and cytotoxicity was observed. Similarly phage was also able to significantly affect all the three parameters in clinical MRSA strains tested for these properties following interaction with phage. These results are in line with the findings of Clem [49] who showed that bacteriophages had protective effect on HEp-G2 cells from cellular damage and apoptosis induced by MRSA isolates.

A combination therapy with antimicrobials differing in their mechanisms of action has been suggested to treat infections. This approach not only provides a broad spectrum of action due to synergistic effect but it also helps in preventing the emergence of drug-resistant subpopulation. It has been proposed that bacteria acquiring simultaneous resistance to both the phage and antibiotic is remote [13],[14],[50]. The results of this study suggest that when used in combination with phage, the frequency of emergence of spontaneous mutants towards mupirocin was effectively decreased to negligible levels (<10^−9^).

To the best of our knowledge, the efficacy of lytic phage in decolonising the nares in an animal model has not been evaluated, though, the efficacy of phage born lytic enzymes has been assessed [51]-[53]. Hence, for assessing the therapeutic potential of phage MR-10 and mupirocin in eliminating the nasal carriage of MRSA 43300, acute nasal colonization model (10 day) was experimentally established in healthy male BALB/c mice. MRSA colonisation was accomplished by putting a stress on the resident flora by increasing the inoculum load (10^6^ CFU/ml, given twice) which helped in the dominance of MRSA 43300 in the nasal tissue over the resident flora. The treatment was started after allowing the bacteria to colonise the nasal tissue of mice (in a period of 48 hours) in order to mimic the scenario prevalent in hospital and community settings, where the treatment is initiated in an already colonised person. Mice receiving two doses of phage MR-10 showed significant reduction (2.8 log cycles) on day 2 itself. Similarly, mupirocin given at a dose of 5 mg/kg (group 3) also showed significant reduction of 2 log cycles on day 2 and minimal bacterial load of 2.2 log CFU/gram on day 7. Both the agents given alone were able to significantly decrease the nasal load of MRSA 43300 by day 7. On the other hand, the group receiving the combined therapy, showed complete clearance of MRSA 43300 in a shorter period of time i.e. by day 5 itself. Also the decrease in bacterial load was significantly greater than the monotherapy groups (group 2 and 3) on all days. Also peak phage titres were observed on day 2 and declined thereafter. In the co-therapy group, phage titres persisted till day 3 only and no plaque was seen on day 5. As phages are highly specific and thus replicate and increase in number at the expense of their respective host bacteria [53],[54] hence no phage activity observed on different days, points towards complete eradication of their host bacteria (MRSA 43300) following treatment with phage. Complete eradication of bacteria was possible due to the combined administration of two agents after allowing successful colonisation of the bacteria in the nasal tissue of mice.

The presence of *S. aureus* in the nose elicits a subclinical immune response, as reported in an earlier study where sero-conversion occurred after carriage was established [55]. Also the host elicits a number of immune factors that constantly impose pressure to eliminate the foreign colonising population [34],[56]. Neutrophils are the most prominent cellular component of the innate immune system and act as an essential primary defence against *S. aureus*[57]. In this study, neutrophil recruitment was studied in terms of MPO levels in all the groups. MPO levels were highest in the untreated colonised group on all post treatment days. The groups receiving phage and mupirocin alone showed peak MPO levels on day 2 and the activity declined to the basal value by day 7. This observation correlates well with the declining bacterial load seen on day 7 in both these groups. Combination therapy group exhibited maximum reduction in MPO levels on day 2 onwards. These results further confirm the efficacy of phages in eliminating the colonized *S. aureus* from the anterior nares of mice. The results of histopathological examination of control (untreated) and treated nasal tissue also substantiated these observations. In the combined therapy group, minimal or no tissue infiltration was seen and the skin of nasal mucosa appeared normal.

The present study indicates that the phage when given along with mupirocin was able to effectively eradicate the colonising population due to their combined action. The dual approach showed maximum nasal protection (better than use of either agent alone i.e. monotherapy) in terms of reduced nasal bacterial load, reduced catalase and MPO levels; with complete elimination of MRSA 43300 occurring by day 5. Coates *et al.*[35] advocated the need to develop potent bactericidal agent than mupirocin on the ground that the newer agents might reduce the relapse rate, clearing the patient of *S. aureus* for a longer period of time than mupirocin. The success obtained with this dual approach is based on the fact that mupirocin being a bacteriostatic antibiotic was able to significantly halt the multiplication and growth of *S. aureus* which was then easily eliminated and cleared off by the lytic phages.

## Conclusion

The present study provides insights into the use of dual therapy for effective decolonisation of MRSA in lesser period of time with reduced chances of relapse and emergence of resistant mutants. In the present study, use of single phage for nasal decolonisation has been looked into, however, for this approach to be successful in clinical settings, need to study a cocktail of phages covering a larger spectrum of strains is required. Also, different delivery systems to achieve a sustained release of the phages may also be investigated.

## Competing interests

The authors declare that they have no competing interests.

## Authors’ contributions

SC, SK: Conceived and designed the experiments; PG: Performed the experiments; SC, SK: Analyzed the data; SC, SK: Wrote the paper. All authors read and approved the final manuscript.

## Additional file

## Supplementary Material

Additional file 1: **Isolation of lytic bacteriophage specific for** ***S. aureus*** **ATCC strains as well as clinical isolates and host range determination.** Click here for file
