# Supplementary material for: Bacteriophage as effective decolonising agent for elimination of MRSA from anterior nares of BALB/c mice
Source: BMC Microbiol. 2014 Aug 12;14:212. doi: 10.1186/s12866-014-0212-8 (PMC4236609; doi:10.1186/s12866-014-0212-8)
Supplement: Additional file 1: — Isolation of lytic bacteriophage specific for S. aureus ATCC strains as well as clinical isolates and host range determination. [file s12866-014-0212-8-S1.docx]

**ADDITIONAL FILE 1 (Manuscript Id: MS: 3414891491099353)**

**Isolation of lytic bacteriophage specific for *S.aureus* ATCC strains as well as clinical isolates and host range determination:**

As listed in Table A1, a total of five lytic bacteriophages were isolated from sewage samples collected from different places in and around Chandigarh. These phages were assigned different numbers (MR-7, MR-8, MR-9, MR-10 and MR-11).

All the five different lytic phages were tested for their host range by spot assay on four standard ATCC strains and 34 clinical isolates. Table A1 shows the lytic spectrum of all the five phages ( MR-7,MR-8 ,MR-9,MR-10 and MR-11).As is clear from the table, phage MR-10 was active against all the four standard strains (*S.aureus* ATCC 43300, *S.aureus* ATCC 29213, *S.aureus* ATCC 33591 and *S.aureus* ATCC 25923) and majority of clinical strains (32/34).

**Table A1:** **Host range of isolated phages**

S.a : *Staphylococcus aureus*,

Different MRSA clinical strains are denoted by *S.a* followed by a number. Same numbers allotted to at time of isolation given by the source (PGIMER) have been retained.

**Isolation of nasal flora and its biochemical identification:**

Three independent colonies were regularly isolated from the nares of randomly selected male BALB/c mice in six independent experiments. These were referred to as NS-1, NS-2 and NS-3.The three different types of isolates were identified on the basis of selected characteristics as presented in Table A2.

Table A2: Preliminary identification of nasal strains isolated from mouse nares (n=6)

| **Nasal strains** | **Gram reaction** | **Colony morphology** | **Hemolysis** | **Catalase** | **Coagulase** | **Growth on Mannitol Salt Agar(MSA)/Mannitol fermentation** |
| --- | --- | --- | --- | --- | --- | --- |
| NS-1 | Gram positive cocci | Cream colored, Pin head | Hemolytic | (+) | (+) | Mannitol fermenting colonies |
| NS-2 | Gram positive cocci | White, small round | No hemolysis | (-) | (ND) | No growth on MSA |
| NS-3 | Gram positive cocci | White, small | No hemolysis | (+) | (-) | Growth on MSA but no mannitol fermentation |

Two strains (NS-1 and NS-3) belonged to genus *Staphylococci* as both the strains were gram positive cocci, catalase positive and fermented glucose in Huge Leifson medium and fermented mannitol.NS-1 coagulated rabbit plasma, hence it was classified as *S.aureus* whereas NS-3 was coagulase negative *Staphylococci*,NS-2 was gram positive cocci in chains, catalase negative and hence belonged to *Streptococcus* group. It was non-hemolytic on blood agar.

**Establishment of nasal colonisation model in BALB/c mice:**

Table A3 depicts the results obtained with bacterial doses i.e 106 and 107 CFU/ml respectively seen on different days post colonisation. The peak counts of *S.aureus* 43300 were seen on day 2 in both the cases. *S.aureus* 43300 persisted well till day 10 post colonisation with a load of 3.98 log CFU/ml (10^6^ CFU/ml) and 4.08 log CFU/ml (10^7^ CFU/ml) respectively. This was followed by a sharp decline of 2 log cycles after which no counts of *S. aureus* 43300 observed on day 15 post colonisation in both the cases.

The total nasal load also followed the same trend, peaking on day 2 followed by gradual decline. A load of 3 log CFU/ml seen on day 12 and 15 accounts for the basal normal flora present. From these observations, it can be concluded that a 10 day nasal colonisation model was established by administering 10^6^ or 10^7^ CFU/ml twice into the anterior nares of BALB/c mice. Since not much difference in the bacterial load of *S.aureus* 43300 was observed with the two doses used in the study, hence 10^6^ CFU/ml was selected for further studies.

**Table A3:** Nasal load of *S. aureus* 43300 as well as total bacterial load in the anterior nares on different days post colonisation in BALB/c mice

| **Days post colonisation** | **Counts in nares following seeding with *S. aureus* 43300** | | | | |
| --- | --- | --- | --- | --- | --- |
|  | **10 ^6^ CFU/ml** | | **10^7^ CFU/ml** | | |
|  | ***S.aureus* 43300** | **Total bacterial load** |  | ***S.aureus* 43300** | **Total bacterial load** |
| **Day 2** | 6.86 ± 0.51 | 7.42 ± 0.12 | **Day 2** | 7.48 ± 0.14 | 9.54 ± 0.31 |
| **Day 5** | 6.24±0.31 | 7.04 ± 0.21 | **Day 5** | 7.01 ± 0.38 | 8.12 ± 0.18 |
| **Day 7** | 5.38 ±0.26 | 6.77 ± 0.22 | **Day 7** | 5.55 ± 0.36 | 7.04 ± 0.23 |
| **Day 10** | 3.98 ± 0.11 | 5.05 ± 0.14 | **Day 10** | 4.08 ± 0.07 | 5.23 ± 0.11 |
| **Day 12** | 2.13 ± 0.015 | 3.91 ± 0.21 | **Day 12** | 2.63 ± 0.22 | 4.14 ± 0.15 |
